# Supplementary material for: RNA processing by the CRISPR-associated NYN ribonuclease
Source: Biochem J. 2024 Jun 17;481(12):793–804. doi: 10.1042/BCJ20240151 (PMC11346440; doi:10.1042/BCJ20240151)
Supplement: Supplementary Material 1 [file BCJ-481-793-s1.pdf]

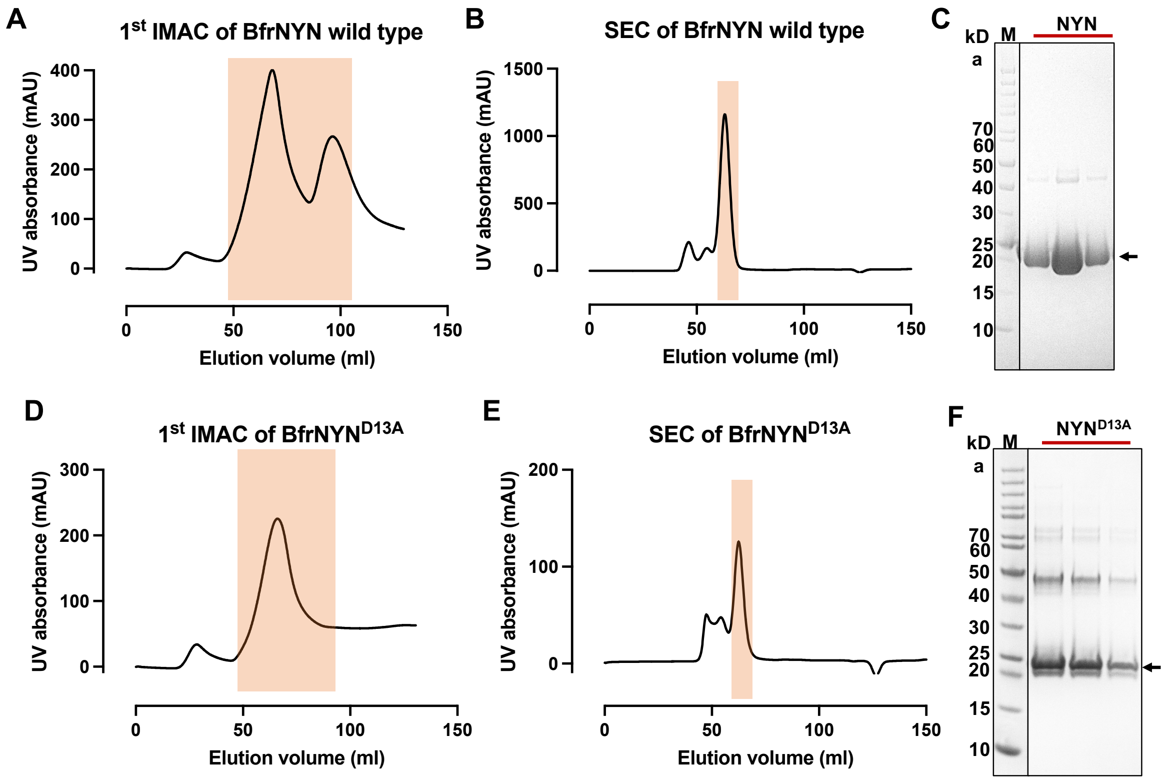

Supplementary Figure 1. The purification of NYN wild type and variant D13A

A, and D. The first immobilised metal affinity chromatography (1st IMAC) step of NYN wild type and variant D13A protein purification. The shaded fractions containing target protein were pooled for poly-histidine tag removal and size exclusion chromatography (SEC) analysis. **B, and E.** SEC profiles. The TEV-cleaved proteins were subjected to SEC. Shaded fractions were collected and concentrated for further enzymatic analysis. **C, and F.** SDS-PAGE analysis of NYN wild type and variant D13A. The predominant species runs at approximately 25 kDa, consistent with the theoretical mass of NYN. M is the marker to indicate size on the gel. A larger species (~48 kDa) was sometimes observed in the SDS-PAGE. This could represent dimeric NYN that was not fully denatured and reduced, but this was not investigated further.

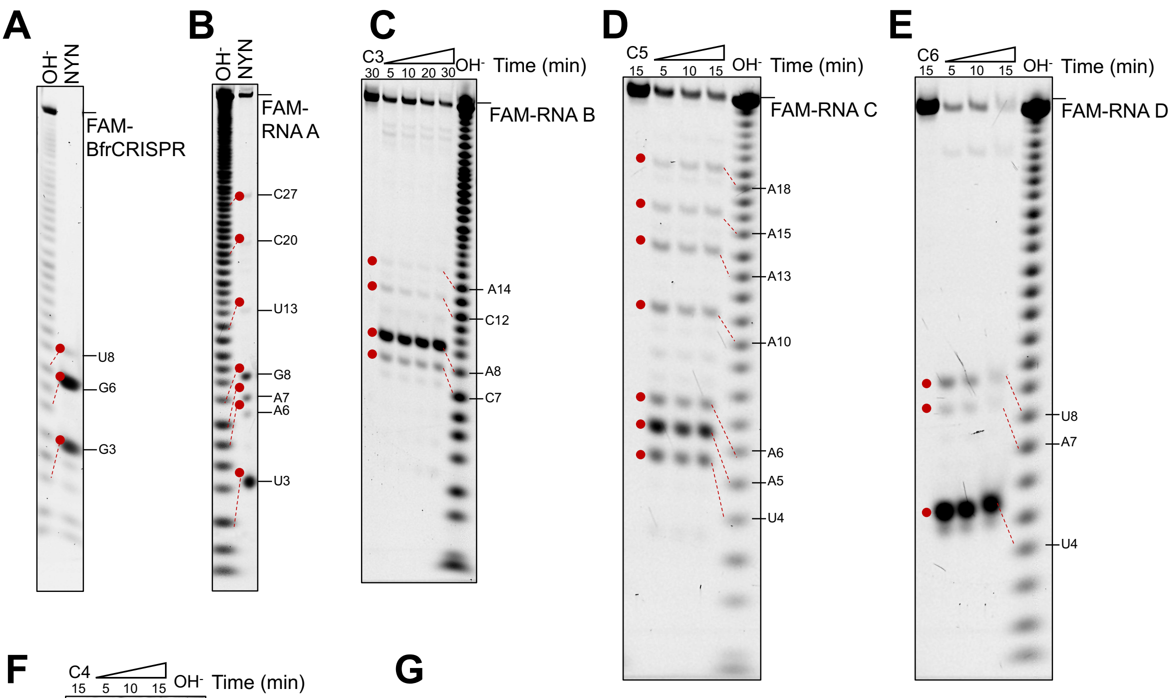

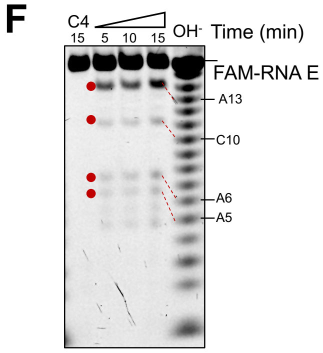

G

RNA	Cleavage sites mapping on the sequence (5' to 3')	Length (nt)
BfrCRISPR	AU**G/**UA**G/**A**U/**GUAUUCCAGUAUAAUAAGGAUUAAGAC	35
A	AU**U/**GA**A/A/G/**ACCA**U/**ACCCAA**C/**UUCUAA**C/**AACGUCGUUCUU AACAACGGAUUAAUCCCAAAA	60
B	UGUCGU**C/A/**GAC**C/**C**A/**AAACCCCGAGAGGGGACGGAAAC	37
C	UGC**U/A/A/**UCU**A/**CU**A/**U**A/**GA**A/**UUGAAAG	25
D	CUU**U/**CA**A/U/**UCUAUAGUAGAUUAGC	24
E	UGAU**A/A**/UCU**C/**UU**A/**UAGA	17

Supplementary Figure 2. Mapping the RNA cleavage sites of NYN.

A-F. Mapping of NYN cleavage sites towards RNA substrates. 5' FAM labelled RNA-BfrCRISPR, A, B, C, D and E were incubated with 200 nM NYN in the presence of Mn^{2+} at 37°C for 30 min or at indicated time points. Cleavage sites were mapped against an alkaline hydrolysis ladder of corresponding RNA species and indicated by red dashed lines. Sequence assignments of bands are shown alongside the gel. The cleaved species indicated by red dots migrate one and half nucleotides slower than bands in the hydrolysis ladder [48], given that the alkaline hydrolysis RNA harbouring a phosphate group at 3' end [49] and Mn^{2+} dependent NYN cleavage products leaving a hydroxyl group at 3' end [50]. **G.** Table of mapped cleavage sites. The cleavage sites were highlighted with red slash and nucleotides indicated alongside the gel were in bold and red.

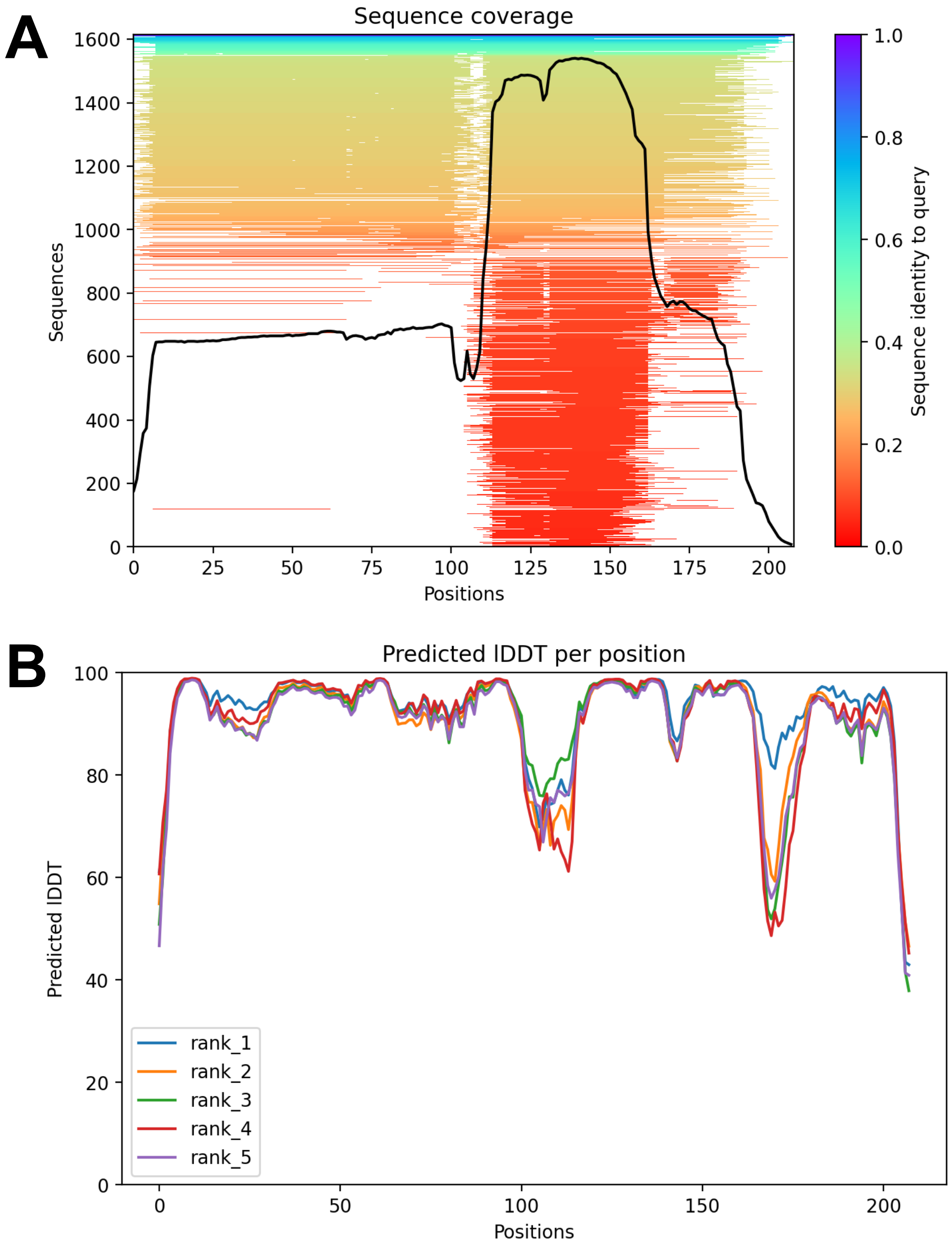

Supplementary Figure 3. Sequence coverage and predicted local distance difference test (pLDDT) scores for the Alphafold2 model of NYN.

A. Heatmap of sequence coverage. All sequences were mapped to the input sequences NYN. The colour scale on the right indicates the identity score, ordered from largest (top) to lowest (bottom). White regions are not covered. The largest coverage of NYN was between 100 and 175. **B. p**LDDT scores per position for the five Alphafold2 models of NYN. Each amino acid position is plotted against the pLDDT values. The pLDDT score for the top ranked NYN model was 92.6, suggesting an accurate model.

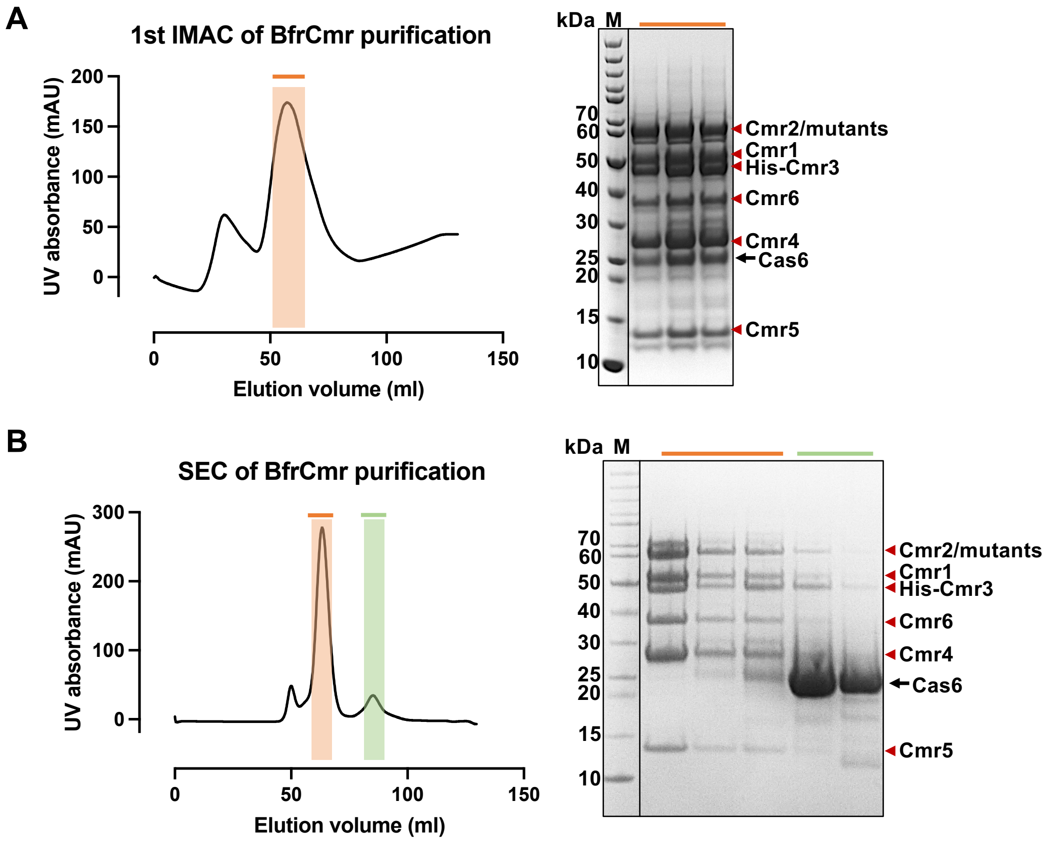

Supplementary Figure 4. Cas6 co-purified with the BfrCmr complex.

A. The first immobilised metal affinity chromatography (1st IMAC) of BfrCmr complex purification. The shaded fractions containing target protein were analysed by SDS-PAGE. Cas6 was co-purified with Cmr complex. **B.** Size exclusion chromatography profile. Fractions pooled from 1st IMAC was subjected to SEC. Shaded fractions were analysed by SDS-PAGE. Cas6 was separated from BfrCmr complex during SEC. The monomer mass of Cas6 is approximately 26 kDa, consistent with its theoretical mass. M is the marker to indicate size on the gel.

Supplementary Table 1 RNA sequences used in this study

**Name**	**Sequence (5'-3')**	**Note**
BfrCRISPR repeat	AUGUAGAUGUAUUCCAGUAUAAUAAGGAUUAAGAC	5' 6-FAM™/35 nt
Target RNA_Lpa (T1)	CACAAGGGACGCCACUUCAUGGAAAUAAAUCACUCACGAAUAGACACGA	RNA/49 nt
Non-target RNA_pUC (T2)	AACGACUCUAGAGGAUCCCCGGGUACCGAGCUCGAAUUCCAAAGGCA	RNA/47 nt
An internally radio-labelled transcript RNA	GGGGAAUUGUGAGCGGAUAACAAUUCCCCUGUAGAAAUAAUUUUGUUUAA<br>CUUUAAUAAGGAGAUAUACCAUGGAAUAGUAAUCUGAUUAUCAAUAUAUG<br>UAGAUGUAUUCCAGUAUAAUAAGG/AUUAAGACAUUCGUGAGUGAUUUAU<br>UUCCAUGAAGUGGCGUCCCUAUGUAGAUGUAUUCCAGUAUAAUAAGG/AU<br>UAAGACUUAAAUAGAGUCGACAAGCUUGCGGCCGCAUAAUGCUUAAGUCG<br>AACAGAAAGUAAUCGUAUUGUACACGGCCGCAUAAUC	T7 transcript/285 nt
RNA A	AUUGAAAGACCAUACCCAACUUCUAACAACGUCGUUCUUAACAACGGAUU<br>AAUCCCAAAA	5' 6-FAM™/60 nt
RNA B	UGUCGUCAGACCCAAAACCCCGAGAGGGGACGGAAAC	5' 6-FAM™/37 nt
RNA C	UGCUAAUCUACUAUAGAAUUGAAAG	5' 6-FAM™/25 nt
RNA D	CUUUCAAUUCUAUAGUAGAUUAGC	5' 6-FAM™/24 nt
RNA E	UGAUAAUCUCUUAUAGA	5' 6-FAM™/17 nt
$PolyU_{10}$	UUUUUUUUUU	3' 6-FAM™/10 nt
